# Microarray-based DNA methylation study of Ewing’s sarcoma of the bone

**DOI:** 10.3892/ol.2014.2322

**Published:** 2014-07-07

**Authors:** HYE-RIM PARK, WOON-WON JUNG, HYUN-SOOK KIM, YONG-KOO PARK

**Affiliations:** 1 Department of Pathology, College of Medicine, Hallym University, Anyang, Gyeonggi 431-070, Republic of Korea; 2 Department of Biomedical Laboratory Science, College of Health Science, Cheongju University, Cheongju, Chungbuk 360-764, Republic of Korea; 3 Department of Biomedical Laboratory Science, College of Health Science, Korea University, Seoul 136-703, Republic of Korea; 4 Department of Pathology, College of Medicine, Kyung Hee University, Seoul 130-702, Republic of Korea

**Keywords:** microarray, DNA methylation, Ewing’s sarcoma

## Abstract

Alterations in DNA methylation patterns are a hallmark of malignancy. However, the majority of epigenetic studies of Ewing’s sarcoma have focused on the analysis of only a few candidate genes. Comprehensive studies are thus lacking and are required. The aim of the present study was to identify novel methylation markers in Ewing’s sarcoma using microarray analysis. The current study reports the microarray-based DNA methylation study of 1,505 CpG sites of 807 cancer-related genes from 69 Ewing’s sarcoma samples. The Illumina GoldenGate Methylation Cancer Panel I microarray was used, and with the appropriate controls (n=14), a total of 92 hypermethylated genes were identified in the Ewing’s sarcoma samples. The majority of the hypermethylated genes were associated with cell adhesion, cell regulation, development and signal transduction. The overall methylation mean values were compared between patients who survived and those that did not. The overall methylation mean was significantly higher in the patients who did not survive (0.25±0.03) than in those who did (0.22±0.05) (P=0.0322). However, the overall methylation mean was not found to significantly correlate with age, gender or tumor location. *GDF10*, *OSM*, *APC* and *HOXA11* were the most significant differentially-methylated genes, however, their methylation levels were not found to significantly correlate with the survival rate. The DNA methylation profile of Ewing’s sarcoma was characterized and 92 genes that were significantly hypermethylated were detected. A trend towards a more aggressive behavior was identified in the methylated group. The results of this study indicated that methylation may be significant in the development of Ewing’s sarcoma.

## Introduction

Ewing’s sarcoma is the second most common type of solid bone and soft-tissue malignancy in children and young adults, and has low cure rates, indicating the requirement to identify further prognostic markers. It has been widely accepted that the acquisition of genetic changes is essential in the development of malignancies. Such alterations include irreversible changes in the DNA sequence, including mutations, translocations, deletions and amplifications, which result in gene activation or inactivation. Epigenetic changes, which represent reversible modifications that affect gene expression without altering the DNA sequence itself, are also a hallmark of cancer (1).

The field of epigenetics describes information transmission through the cell division of heritable changes in a phenotype that does not involve DNA sequence changes and is transferred by cell division. CpG island hypermethylation, histone modification and transmitted chromatin structures are mechanisms underlying epigenetic transmission, and among these, CpG island hypermethylation is a key component for altered gene expression associated with human cancer (2). Although the causes are unclear, promoter CpG island hypermethylation may be associated with aging and cancer development (2). Promoter CpG island hypermethylation is found in virtually all human cancer tissue types and acts as an important mechanism for the inactivation of tumor suppressor and tumor-related genes (3).

Aberrant DNA methylation has been recognized as an early event in tumorigenesis (4,5) and therefore, variations in the methylation patterns identified between normal and tumor cells may aid in the detection of tumor cells in biopsy specimens or tumor-derived DNA in body fluids (6). The advent of high-throughput microarray technology allows for the simultaneous evaluation of genome-wide DNA methylation patterns and RNA expression levels in tumor specimens, and also allows for the identification of molecular targets or gene classifiers that are specific to tumor cells (7).

The number of genes demonstrated to be inactivated by promoter CpG island hypermethylation has abruptly increased with the application of array-based genome-scale DNA methylation analysis. The GoldenGate assay for methylation has successfully analyzed the methylation profiles of 1,536 CpG sites from 371 genes identified in cancer cell lines, lung cancer and normal tissues, and has identified a panel of markers specific for adenocarcinoma methylation (8,9). The assay has also been used to assess the epigenetic specificity of the loss of *IGF2* imprinting in Wilms’ tumors and to identify the unique epigenetic signature of human embryonic stem cells (10,11). However, there is little information concerning similar broad-based studies of Ewing’s sarcoma*.* The objective of the present study was to analyze methylation patterns and to assess their clinical significance in Ewing’s sarcomas.

## Materials and methods

### Ewing’s sarcoma samples and controls

This study was approved by the Institutional Review Board of Kyung Hee University Hospital (Seoul, Korea). A total of 69 samples from patients with Ewing’s sarcomas were analyzed. The disease was diagnosed based on the World Health Organization criteria (12). Briefly, Ewing’s sarcoma is a small round cell sarcoma, with diffuse membranous CD99 immunostaining, cytoplasmic periodic acid-Schiff staining and *EWSR1* gene translocation, as determined using Zytolight SPEC *ROS1* and *RET* Dual color break apart probes and demonstrated with fluorescence *in situ* hybridization, according to the manufacturer’s instructions (Zytovision, Bremerhaven, Germany). The clinical features of the patients are summarized in Table I. The age at diagnosis ranged between one and 57 years, and 39 patients were male and 30 were female. The primary sites were the long bones (n=31) and the flat, small bones and spine (n=38). Metastasis at diagnosis was present in five patients. The follow-up data were only available for 37 patients and the follow-up durations ranged between six and 240 months. As control samples, 14 tissue specimens were used that were obtained from the cancellous bone during total hip or knee joint replacement surgeries due to degenerative osteoarthritis. These included the marrow components of the tibia or femur.

### Preparation of DNA samples

DNA extraction was performed as described previously (13). Genomic DNA was extracted from the formalin-fixed, paraffin-embedded (FFPE) tissue sections of each sample by a Magna Pure LC instrument (Roche Diagnostics GmbH, Mannheim, Germany). Briefly, 10-μm paraffin sections were mixed gently with 800 μl xylol and 400 μl absolute ethyl alcohol by inverting the tube several times. The supernatant was discarded following brief centrifugation at 12,000 × g for 10 mins, and the pellet was washed with 1 ml absolute ethyl alcohol. The pellet was dried for 10 min at 55°C following removal of the supernatant. The tissue pellet was vortexed with 80 μl of a tissue lysis buffer (Roche diagnostics GmbH) and 20 μl proteinase K, followed by overnight incubation at 55°C. The digested samples were loaded into the Magna Pure LC instrument. All DNA samples were stored at −70°C prior to use. The DNA concentrations derived from the FFPE samples were determined on a fluorophotometer (Victor3; Perkin-Elmer, Waltham, MA, USA), using the PicoGreen nucleic acid quantification kit (PicoGreen; Molecular Probes, Eugene, OR, USA), which allows accurate and reproducible DNA quantification at low concentrations, including DNA extracted from archival FFPE samples (14).

### Bisulfite conversion and methylation chip assay

Bisulfite conversions of all DNA samples were performed using an EZ-96 DNA methylation kit (Zymo Research Corporation, Orange, CA, USA) according to the manufacturer’s instructions. In total, 500 ng of genomic DNA was used for each bisulfite conversion. Following bisulfite treatment, the quantification of the methylcytosine content was performed using the Illumina GoldenGate Methylation Cancer Panel I microarray, as described previously (3). The GoldenGate Methylation Cancer Panel I product was used to process 1,505 CpG sites from a panel of 807 cancer-related genes, which included oncogenes and genes associated with DNA repair, tumor suppression, cell cycle, differentiation and apoptosis. Of these 1,505 CpG sites, 1,044 were located within CpG islands and 461 were located outside CpG islands. Briefly, the bisulfite-converted DNA was reacted with biotin and hybridized to assay oligos. Next, specific extensions and ligations were performed at 45°C for 15 min. The ligated products were amplified by polymerase chain reaction (PCR) and conditioned as follows: 10 min at 37°C; then 34 cycles of 35 sec at 95°C, 35 sec at 56°C, 2 min at 72°C; 10 min at 72°C; and cooling for 5 min at 4°C. Single-stranded PCR products were prepared by denaturation and hybridized to a Sentrix Array Matrix (GoldenGate Methylation Cancer Panel I). The array hybridization was conducted overnight under a temperature gradient program ranging from 45 to 60°C, and arrays were imaged using a Bead Array Reader scanner (Illumina, San Diego, CA, USA). The raw methylation ratios were calculated using the Illumina BeadStudio Methylation Module (Illumina) following background normalization, which was derived by averaging the signals of the built-in negative control (15). Each sample was examined in a duplicate manner in the chip assay.

### Statistical analysis

Data were analyzed using SAS version 4 (SAS Institute, Cary, NC, USA). The two-sample t-test and a one-way analysis of variance were performed to estimate the methylation profiles on clinical parameters and survival rate. P<0.05 was considered to indicate a statistically significant difference.

## Results

**Figure 1:**
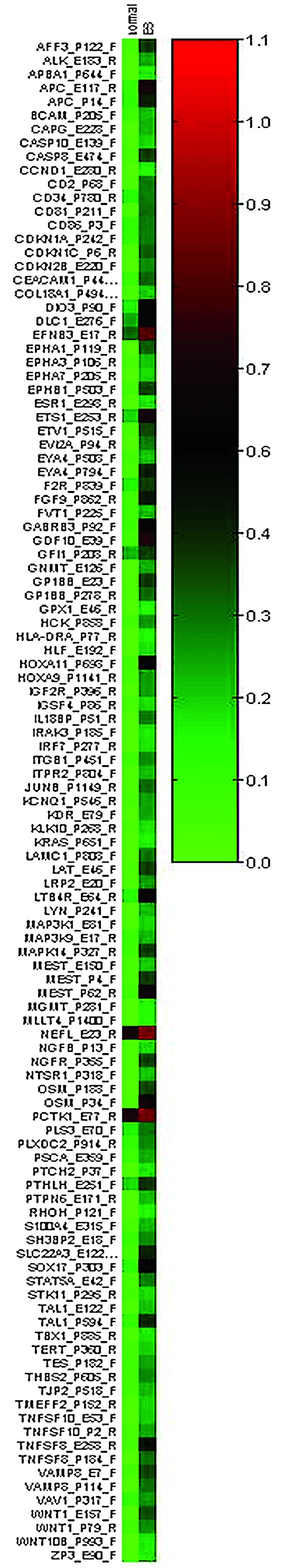
Heat map showing the differentially-methylated CpGs in the Ewing’s sarcoma samples. Red, methylated genes; green, unmethylated genes.

The DNA methylation status of 69 Ewing’s sarcoma samples was examined using the Illumina GoldenGate Methylation Cancer Panel I microarray. The GoldenGate DNA methylation assay measures the DNA methylation levels of a given locus as β-values ranging from 0 (no DNA methylation detected) to 1 (complete DNA methylation). The methylation patterns of the Ewing’s sarcoma samples were extremely heterogeneous with respect to total DNA methylation. The criteria for differentially-methylated genes were applied to detect the genes whose DNA methylation levels (β) differed by at least 0.15 between the Ewing’s sarcoma and control samples (8). Therefore, a list of 92 differentially-methylated genes was obtained from the Ewing’s sarcoma samples. The 92 genes were classified into nine groups based on their biological functions: i) Cell adhesion (n=10); ii) cell cycles (n=4); iii) cell regulation (n=22); iv) development (n=13); v) immune response (n=4); vi) metabolism (n=4); vii) protein regulation (n=5); viii) signal transduction (n=25); and ix) transcription regulation (n=5). A list of the methylated genes identified in Ewing’s sarcoma is shown in Table II. Fig. 1 shows a heat map of the differentially-methylated CpGs in the Ewing’s sarcoma samples.

The overall methylation mean of each tumor was compared according to survival status. The overall methylation mean was significantly higher in the patients who did not survive (0.25±0.03) compared with the surviving patients (0.22±0.05) (P=0.0322). However, no significant correlation was identified between the overall methylation mean and the clinical parameters of age, gender and tumor location (Table I).

Using a highly stringent selection criteria (a β difference of <0.5), four unique genes (*GDF, OSM, APC* and *HOXA11*) were selected that were the most significantly differentially-methylated genes in the Ewing’s sarcoma samples. The methylation of these top four genes was confirmed to be common, occurring in 82.5% (*GDF*), 65% (*OSM*), 87.5% (*APC*) and 45% (*HOXA11*) of the Ewing’s sarcoma samples. However, their methylation levels were not found to significantly correlate with the survival rate (Table III).

## Discussion

Ewing’s sarcoma is comprised of morphologically heterogeneous tumors that are themselves characterized by non-random chromosomal translocations of the *EWS* gene and one of the members of the ETS family of transcription factors. The (11;22)(q24;q12) translocation is the most frequently occurring, and results in the formation of the EWS-FLI1 fusion protein. This protein aids Ewing’s sarcoma pathogenesis by modulating target gene expression (16). However, only a few studies have analyzed gene methylation in Ewing’s sarcoma*.* These studies have reported that the hypermethylation of *HIC1, MGMT, CDH1, p15* and *p16* in tumors, as well as the hypermethylation of *CASPASE 8,* occurs only in Ewing’s sarcoma cell lines (6,17–19). The number of genes that have been shown to be inactivated by promoter CpG island hypermethylation has abruptly increased with the application of array-based genome-scale DNA methylation analysis. However, there is little information concerning similar broad-based methylation studies on Ewing’s sarcoma. The main goal of the present study was to provide a general overview of the changes in DNA methylation associated with Ewing’s sarcoma*.*

The GoldenGate Cancer Panel I used in this study offers the ability to analyze 1,505 single CpG loci corresponding to 807 genes in parallel. The reproducibility and accuracy of this array-based approach have been extensively demonstrated (15,20). Using the aforementioned criteria, 92 differentially-hypermethylated genes were identified in Ewing’s sarcoma. These included numerous genes known to affect tumorigenesis by affecting cell regulation, signal transduction and differentiation. These results demonstrated that the GoldenGate assay offers a high-throughput method to identify novel genes with promoter DNA methylation.

Promoter CpG island hypermethylation can be used as a tumor biomarker that is able to detect tumor cells in serum or to predict clinical outcome. The simultaneous hypermethylation of multiple CpG island loci, particularly the CpG island methylator phenotype (CIMP), may be associated with survival rather than individual gene hypermethylation. The widespread hypermethylation of multiple promoter CpG island loci characterizes a subset of malignancies, designated as CIMP (2). The clinicopathological features of CIMP-positive Ewing’s sarcoma remain obscure, and marker panels for diagnosing CIMP-positive Ewing’s sarcoma have not yet been established.

The overall methylation mean of the tumor samples was compared with survival rate, and the overall methylation mean was significantly higher in the patients who did not survive compared with those who did. A trend towards a more aggressive behavior was identified in the methylated samples. However, no significant correlation was identified between the overall methylation mean and the clinical parameters of age, gender and tumor location. Thus, four unique genes (*GDF10*, *OSM, APC,* and *HOXA11*) were selected that were the most significantly differentially methylated in the Ewing’s sarcoma samples. The top four hypermethylated genes may function as differential epigenetic biomarkers between Ewing’s sarcoma and control samples. However, their methylation levels were not found to significantly correlate with the survival rate.

Among these genes, *GDF10* has been reported to have a gender-dependent effect on glioblastoma progression and survival (21). The location of the *OSM* gene has been found to be distal to the translocation breakpoint on chromosome 22 of Ewing’s sarcoma (22). Higher levels of *OSM* have also been reported in metastasizing prostate cancer compared with non-metastasizing prostate cancer and benign prostatic hyperplasia (23). Furthermore, APC promoter hypermethylation has been shown to be an early event in endometrial tumorigenesis (24). *HOX* genes are important members of the homeobox superfamily, encoding transcription factors and contributing to oncogenesis through the activation of anti-apoptotic pathways (25). Fiegl *et al* (26) revealed that *HOXA11* gene DNA methylation frequently occurs in ovarian cancer and that consequently, HOXA11 methylation status is a prognostic marker.

Avigad *et al* (27) analyzed the aberrant methylation of *RASSF1A* in Ewing’s sarcoma using methylation-specific PCR. The study stated that Ewing’s sarcoma patients with methylated *RASSF1A* showed poorer prognoses than those without. However, contrasting results were identified in the current study. In addition, Harada *et al* (28) reported that there is no methylation of *RASSF1A* in Ewing’s sarcoma*.* These discordant results may be due to differences in the detection method or in the study population.

The results of the present study not only provide novel insights into the biology of Ewing’s sarcoma, but also have potential therapeutic implications. DNA methylation inhibitors, such as decitabine and 5-azacitidine, are currently used in clinical studies to treat myelodysplastic syndrome or acute myeloid leukemia patients (29). If DNA methylation inhibitors exert therapeutic effects on the demethylation of hypermethylated genes, Ewing’s sarcoma with a higher level of DNA hypermethylation may theoretically be a better target for such drugs. Thus, DNA methylation profiling may be a useful approach to monitoring the association between epigenetic and clinical responses, and to stratify patients for treatment with demethylating agents.

In conclusion, the current study identified the hypermethylation of 92 genes in Ewing’s sarcoma and a trend toward more aggressive behaviors in samples with methylation. The top four hypermethylated genes can be used as differential epigenetic biomarkers for Ewing’s sarcoma. However, further studies on their clinical implications with a larger scale sample are required to confirm these results.

## Figures and Tables

**Table I:** Overall methylation mean values of Ewing’s sarcoma samples according to the clinical parameters.

Parameter	Overall methylation mean	P-value
Age, years		0.3659
<20 (n=46)	0.23±0.04	
≥20 (n=23)	0.24±0.04	
Gender		0.3304
Male (n=39)	0.24±0.04	
Female (n=30)	0.23±0.04	
Location		0.0761
Long bone (n=31)	0.23±0.04	
Flat, small bone, spine (n=38)	0.24±0.04	
Survival		0.0322^a^
Not alive (n=18)	0.25±0.03	
Alive (n=19)	0.22±0.05	

a Statistically significant.

Overall methylation mean data are presented as the mean ± standard deviation.

**Table II:** List of methylated genes in Ewing’s sarcoma.

Function	Genes
Cell adhesion (n=10)	APC, GP1BB, LAMC1, CD2, THBS2, ITGB1, COL18A1, APBA1, CD34, BCAM
Cell cycle (n=4)	CDKN, KLK10, PCTK1, KRAS
Cell regulation (n=22)	OSM, TNFSF8, ETS1, EFNB3, LTB4R, FGF9, NGFR, WNT1, CASP8, TAL1, MAPK14, DLC1, KDR, CEACAM1, LYN, PTPN6, F2R, TNFSF10, PTHLH, CD86, CASP10, ESR1
Development (n=13)	GDF10, MEST, AFF3, EPHB1, ALK, HOXA9, HLF, PLXDC2, PTCH2, WNT10B, PSCA, ZP3, TBX1
Immune response (n=4)	IL18BP, STAT5A, IRF7, HLA-DRA
Metabolism (n=4)	EYA4, LRP2, FVT1, GPX1
Protein regulation (n=5)	DIO3, VAMP8, GNMT, CAPG, TJP2
Signal transduction (n=25)	GABRB3, SLC22A3, LAT, EPHA1, GP1BB, EVI2A, CD81, IGF2R, KCNQ1, HCK, NTSR1, ITPR2, S100A4, SH3BP2, EPHA7, EPHA3, TES, NEFL, TMEFF2, MAP3K1, VAV1, NGFB, IRAK3, RHOH, STK11
Transcription regulation (n=5)	HOXA11, SOX17, ETV1, JUNB, TERT

**Table III:** Methylation profiles of four unique genes in Ewing’s sarcoma samples according to survival rate.

Gene	Not alive (n=18)	Alive (n=19)	P-value
GDF10	0.53±0.34	0.52±0.35	0.4528
OSM	0.46±0.38	0.44±0.37	0.4475
APC	0.51±0.35	0.55±0.28	0.3305
HOXA11	0.29±0.37	0.38±0.40	0.2336

Data are presented as the mean ± standard deviation.
